# In-office arthroscopy for the evaluation of chronic knee pain: A case report

**DOI:** 10.1177/2050313X17740992

**Published:** 2017-11-14

**Authors:** Jacob A West, Nirav H Amin

**Affiliations:** Loma Linda University, Loma Linda, CA, USA

**Keywords:** Sports medicine, arthroscopy, meniscal tear

## Abstract

This is a case report detailing the use of in-office needle arthroscopy (mi-eye 2™) in a patient with chronic knee pain and inconclusive magnetic resonance imaging findings. The patient is a 40-year-old male who presented to our clinic after an extended history of right knee pain along the medial aspect with previous failed treatments. Magnetic resonance imaging without contrast had demonstrated full-thickness chondral fissuring of the lateral patellar facet, mild abnormal signals of the proximal patellar tendon and Hoffa’s fat pad, and intact anterior cruciate ligament and posterior cruciate ligament. The patient was previously treated with an ultrasound-guided injection of 2 cm^3^ of 1% lidocaine without epinephrine and 1 cm^3^ of Kenalog-40 and scheduled for follow-up. At follow-up, clinical examination showed antalgic gait, minimal tenderness along medial joint line, medial pain in deep flexion, and no pain when in varus or valgus. Due to continued discomfort with a negative magnetic resonance imaging, in-office diagnostic arthroscopy was performed using mi-eye 2 revealing a tear of the mid-body of the medial meniscus. The patient subsequently underwent arthroscopic repair and is recovering well with complete resolution of medial joint pain. This report highlights the clinical utility of in-office diagnostic arthroscopy in the management of patients with persistent knee pain and negative or equivocal findings on magnetic resonance imaging.

## Introduction

Knee pain is among the most common issues that orthopedic surgeons diagnose and treat. Advanced imaging, including magnetic resonance imaging (MRI), is frequently obtained to aid in the diagnosis. Despite the superior soft tissue resolution of MRI, it is not infallible. In a study of the diagnostic accuracy of MRI for meniscal tears, the specificity was cited as 93% for acute tears and 91% for chronic tears. In contrast, the sensitivity of MRI was only 67% for acute traumatic tears and 64% for chronic tears.^1^ This suggests that although positive findings on MRI are highly suggestive of meniscal injury, a negative MRI does not fully exclude meniscal injury in the setting of high clinical suspicion. Other data suggest that MRI has poor diagnostic accuracy in identifying tears of the posterior horn of the lateral meniscus (PHLM) especially in cases of concomitant anterior cruciate ligament (ACL) injury. In a retrospective analysis of 120 patients who underwent arthroscopic ACL reconstruction, 28 meniscal tears were missed on MRI. Of these, 19 (67%) were in the PHLM.^2^ In a prospective cohort study of 65 patients with chronic ACL tear, the sensitivity of MRI was found to be significantly lower in detecting isolated tears of the posterior horns of the meniscus, while tears involving the anterior horn were rarely missed.^3^ Thus, due to the prevalence of false-negative results on MRI, diagnostic arthroscopy remains the gold standard for diagnosis of meniscal injury when MRI is negative or equivocal. Diagnostic arthroscopy is not a completely benign procedure, however. Placing a patient under general anesthesia and entering the knee capsule poses a variety of risks. In a retrospective analysis of 92,565 arthroscopic knee surgeries, Salzler et al.^4^ noted 4305 complications (4.7%). The most common complication was infection which had an incidence of 0.84%. An alternative diagnostic option exists, however, which allows the surgeon to visualize the compartments of the knee in an in-office setting. In-office needle arthroscopy avoids the risk of anesthesia, delivers immediate answers to the patient’s symptoms, and provides cost savings to the patient, hospital, and insurance company. We present a patient with persistent right knee pain and previous inconclusive examinations and MRI studies. The patient was informed that data concerning his case would be submitted for publication, and written consent was obtained.

## Case report

The patient, a 40-year-old male, presented to our clinic with a history of right knee pain predominately along the medial aspect. His medical history includes prior examinations and treatments by another physician for right knee pain which provided minimal relief. The patient’s pain originated 2 months prior, wherein he experienced moderate pain that became sharp when squatting and bending, as well as stiffness while sleeping. The pain subsided with extension of the extremity, but loading of the joint re-aggravated the pain. Physical examination showed good range of motion (ROM), slight patellofemoral crepitus, and no meniscal signs along the medial joint line.

MRI with no contrast was obtained, which demonstrated full-thickness chondral fissuring of the lateral patellar facet, mild abnormal signals of the proximal patellar tendon and Hoffa’s fat pad, and intact ACL and posterior cruciate ligament (PCL). The patient was treated with an ultrasound-guided injection of 2 cm^3^ of 1% lidocaine without epinephrine and 1 cm^3^ of Kenalog-40. He experienced immediate pain relief in his knee and was scheduled for clinical follow-up in 6 weeks.

The patient first presented to our office approximately 6 weeks following last examination and treatment from previous physician. He presented with medial pain of the right knee that was sharp but minor in nature. The pain was predominately activity-related and the patient was able to ambulate on his own. The patient stated that he was active as a bodybuilder and was a professional fighter for the prior 12 years. Our physical examination showed an antalgic gait with the patient favoring the affected extremity. The patient also exhibited minimal focal tenderness below the medial joint line and a negative McMurray test. Patient’s ROM was 0–120°+ and the knee was stable in reverse pivot and varus and valgus stress. When positioned in deep flexion, the patient experienced pain along the medial side of the knee. I personally reviewed the previous MRI the patient received while under previous physician’s care. Upon examination and imaging review, the patient was prescribed a lidocaine cream for pain management, and Pilates for stretching exercises were recommended. The patient was subsequently scheduled for follow-up in 6 weeks.

The patient returned to our office for 6-week follow-up visit. Approximately 2 weeks prior, he had experienced an injury to his right knee while teaching a kickboxing class, which had caused severe pain. The pain was severe enough in nature to cause difficulty and discomfort while sleeping. He had applied the previously prescribed lidocaine for the pain, but it offered only little relief to the knee. A physical examination was performed, which resulted in similar results as the previous examination—antalgic gait, minimal tenderness along medial joint line, medial pain in deep flexion, and no pain when in varus or valgus. We discussed the patient’s injury history and it was our recommendation to perform an in-office diagnostic arthroscopy (mi-eye 2™) due to the continued pain and discomfort with a negative MRI. The patient agreed with this recommendation and was scheduled to return for mi-eye 2 procedure in approximately 2 weeks with the goal to visualize pathology and develop a treatment plan.

**Figure 1.**
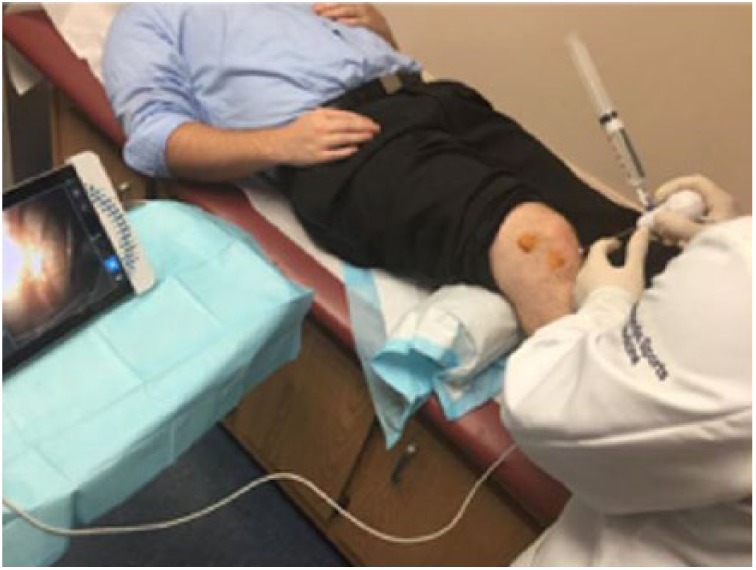
In-office image of the mi-eye 2™ arthroscope being used to evaluate the knee.

**Figure 2.**
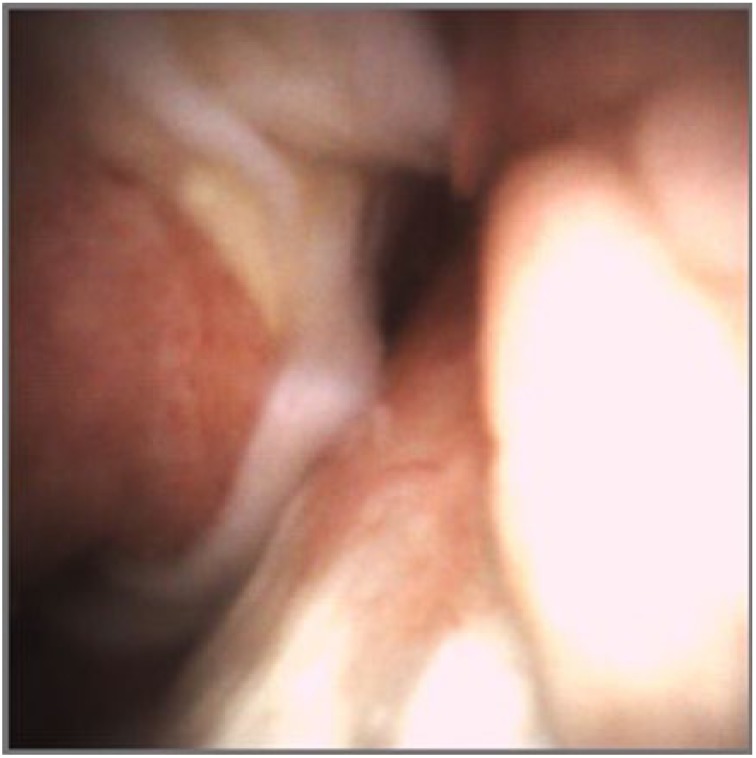
Intact anterior cruciate ligament visualized ascending into the femoral notch using in-office arthroscopy (mi-eye 2™).

**Figure 3.**
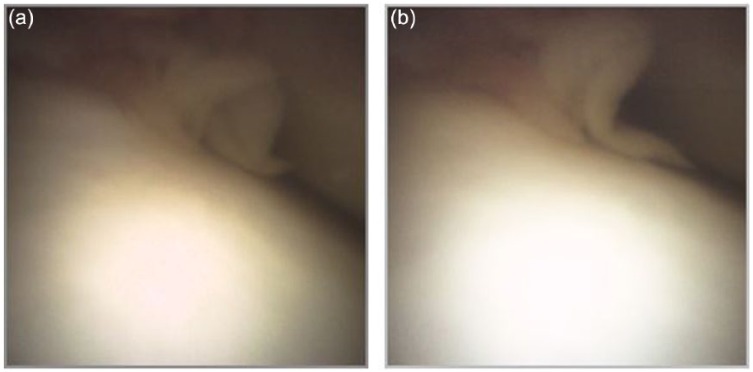
(a) Visualization of the mid body tear of the medial meniscus through in-office diagnostic arthroscopy; (b) Another image showing the tear from a different angle.

Upon return for the in-office arthroscopy, the patient was positioned lying down with a “bump” placed under the right knee for flexion (Figure 1). The procedure was performed in a sterile environment, and the knee portals were aseptically prepared. The patient was given an analgesic injection of 1% lidocaine (5 cm^3^). The mi-eye 2 was inserted into the right knee and immediately—within approximately 20 s—the intact ACL was visualized (Figure 2) and a tear of the mid-body of the medial meniscus was identified (Figure 3(a) and (b)). Through the duration of the procedure, minimal saline was needed—approximately 4 cm^3^. Following visualization, we discussed the treatment options with the patient, who ultimately opted for surgical intervention.

**Figure 4.**
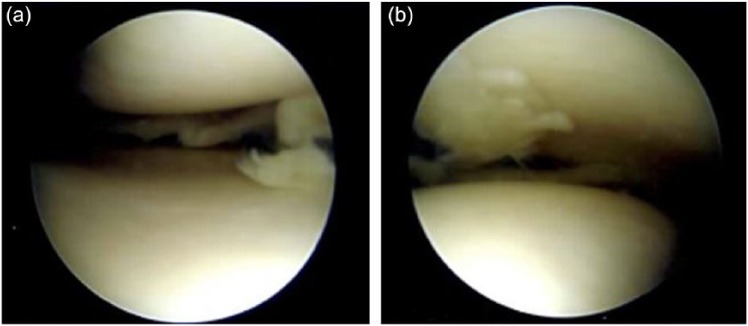
(a) Image taken during arthroscopy under general anesthesia confirming mid-body tear of medial meniscus previously visualized using in office diagnostic arthroscopy; (b) Another image showing the tear from a different angle.

**Figure 5.**
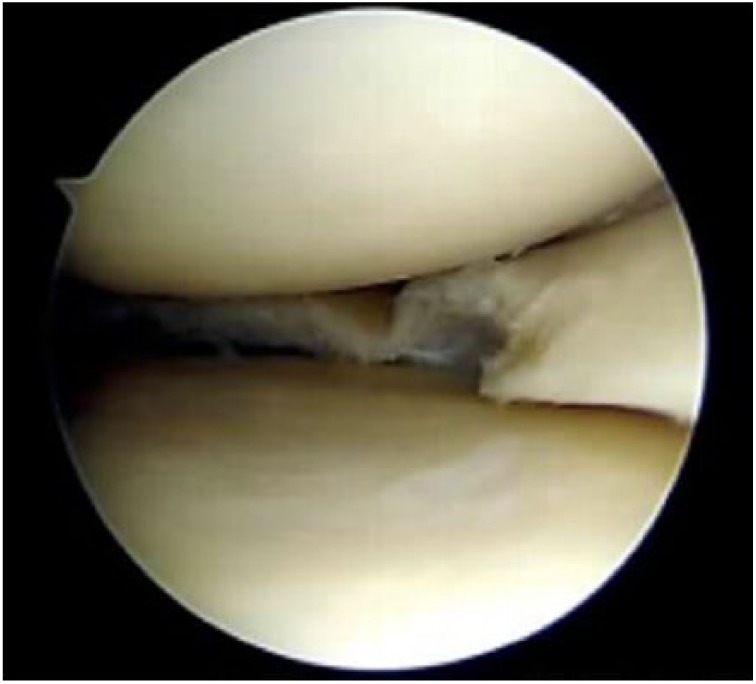
Image of the meniscus after debridement to a stable rim.

The patient was scheduled for surgery 2 days later to address the meniscus tear visualized with mi-eye 2. In addition to the mid-body tear of the medial meniscus, surgical findings included grade II changes to the medial and lateral edges of the patella and radial tear of the posteromedial aspect of the medial meniscus. The mid-body meniscal tear previously visualized using mi-eye 2 was confirmed (Figure 4(a) and (b)) and the meniscus was debrided to a stable rim (Figure 5). The patient was discharged following recovery from anesthesia and allowed for weight-bearing on his right knee as tolerated. He was scheduled for follow-up 3 weeks post-operatively.

Following 2 weeks of surgery, the patient reported good functional motility and resolution of medial-sided joint pain.

## Discussion

Knee injuries are very common in athletes and active individuals with a significant percentage involving the menisci. Annually, more than 950,000 arthroscopic surgeries are performed in the United States on the knee alone.^5^ Of these surgeries, nearly half are related to medial and/or lateral meniscus injuries, with annual direct medical costs estimated at US$4 billion.^6,7^ MRI is commonly used to aid in the diagnosis of internal derangement of the knee due to its superior soft tissue resolution. With reported accuracy rates of 90% or greater, the results of MRI frequently play a role in determining surgical or conservative management for patients.^8,9^

Some studies have questioned the ability of MRI to accurately detect and characterize the size of articular cartilage defects. A study comparing MRI reports from musculoskeletal radiologists to arthroscopic findings in 82 patients found that MRI reports missed 55% of all chondral lesions.^10^ Gomoll et al. investigated the disparity between intra-operative measurements of chondral defect size and preoperative MRI size estimates in 37 patients who underwent open cartilage repair. They found that 85% of all defects were larger than predicted on MRI by an average of 65%, and only 8% of defects were accurately predicted (within 10% of final size).^11^ In a similar study of 77 patients, Campbell et al.^12^ found that 74% of defects were larger than MRI estimates, which underestimated the size by 70% on average. These findings have important implications as treatment algorithms in cartilage repair are based primarily on defect size, and reliance on preoperative MRI scans alone has the potential to compromise treatment decisions.

The literature also suggests that the diagnostic utility of MRI may be decreased in certain situations. In a retrospective analysis of 400 patients, De Smet and Graf^13^ showed that in the presence of a tear of the ACL, the sensitivity of MRI decreased from 0.97 to 0.88 for medial meniscal tears and from 0.94 to 0.69 for lateral tears. In a similar study of meniscal tears by Nam et al.,^14^ the negative predictive value of MRI for ruling out meniscal injury was significantly decreased when there was a concomitant injury to the ACL. A meta-analysis of 14 studies on the diagnostic utility of MRI in meniscal tears showed that although the sensitivity for diagnosing medial meniscal tears was relatively high using MRI (89%), the sensitivity was significantly decreased in diagnosing lateral meniscal tears (78%).^9^ These studies suggest that there may be certain meniscus pathologies which are not as readily detected by MRI, and alternative diagnostic techniques may be appropriate. Additional considerations pertaining to the use of MRI include patients who are unable to obtain an MRI (i.e. due to a pacemaker, aneurysm clips, severe claustrophobia) as well as the relatively high associated cost. As high deductible insurance plans gain widespread adoption, out-of-pocket costs for elective outpatient MRI may become prohibitive. In a national survey on the cost of knee MRI in the United States, Pasalic et al. found that costs ranged from US$259 to US$2042 across all centers. The median out-of-pocket costs of knee MRI for the West, Northeast, Midwest, and South regions were US$690, US$500, US$550, and US$550, respectively.^15^ In contrast, the total direct cost of in-office needle arthroscopy as indicated by CY 2013 Medicare reimbursement data was $603, leading to significantly lower out-of-pocket costs for patients.^16^ In a recent retrospective review of 175 patients undergoing in-office needle arthroscopy of the knee, McMillan et al. found that the average reimbursement was US$628.92 (range: US$340–US$1391). This was significantly less than the outpatient cost of MRI which averaged US$1047 (range: US$565–US$2100).^17^ There is undoubtedly a role for an alternative diagnostic modality, which may mitigate some of the issues mentioned above.

Small-bore (needle) arthroscopy represents an alternative diagnostic tool to assist in obtaining an accurate and timely diagnosis. Needle arthroscopy has shown to be a safe and effective means of obtaining direct visualization of a joint.^18,19^ The mi-eye 2 is a 2.2-mm arthroscope, consisting of a needle, integrated camera, and light source, combined in a single-use device. The images are displayed on a high-definition tablet, which provides convenient portability. With this device, we were able to obtain an immediate and definitive diagnosis in a situation that would have otherwise required a formal diagnostic arthroscopy in the operating room, thus saving the patient a general anesthesia event. The mi-eye 2 greatly expedited the time to diagnose and treat our patient’s pathology. Additionally, visualizing the previously uncertain and unexpected pathology prior to the operating room allowed us to appropriately adjust the surgical plan, discuss treatment expectations and outcomes with the patient, and have the necessary instruments available during surgery.

A timelier and more definitive diagnosis and treatment plan, combined with fewer office visits and decreasing potentially unnecessary diagnostic studies and surgeries, can result in a significant reduction in health care costs. In fact, the use of in-office arthroscopy in place of MRI for patients presenting with meniscal pathology was reported to result in a net cost savings of US$151 million annually.^16^ The ability to directly visualize inside a patient’s joint while they are awake provides the patient the opportunity to view and review the images in real time and be actively involved in their diagnosis and treatment. This can result in an improved patient experience and help foster a healthy relationship between the patient and the surgeon. Ultimately, in-office arthroscopy can be a very valuable tool in the diagnosis and treatment of intra-articular pathology, providing distinct benefits for both the orthopedic surgeon and the patient, as highlighted in our care of this patient.

## Conclusion

As evidenced in this case report, in-office needle arthroscopy is quick and cost-effective making it a valuable adjunctive tool in the diagnosis of internal derangements of the knee.
